# Processes of believing: Where do they come from? What are they good for?

**DOI:** 10.12688/f1000research.9773.2

**Published:** 2017-01-17

**Authors:** Rüdiger J. Seitz, Raymond F. Paloutzian, Hans-Ferdinand Angel

**Affiliations:** 1 Heinrich-Heine-University Düsseldorf, LVR-Klinikum Düsseldorf, Düsseldorf, Germany; 2 Westmont College, St. Barbara, USA; 3 Institute of Catechetic and Pedagogic of Religion, Karl Franzens University Graz, Graz, Austria

**Keywords:** Belief, belief systems, behavior, credition, cerebral networks, meaning making, narratives, rituals, representations, perception, prediction, religion, valuation

## Abstract

Despite the long scholarly discourse in Western theology and philosophy on religion, spirituality, and faith, explanations of what a belief and what believing is are still lacking. Recently, cognitive neuroscience research addressed the human capacity of believing. We present evidence suggesting that believing is a human brain function which results in probabilistic representations with attributes of personal meaning and value and thereby guides individuals’ behavior. We propose that the same mental processes operating on narratives and rituals constitute belief systems in individuals and social groups. Our theoretical model of believing is suited to account for secular and non-secular belief formation.

## Summary

Although largely neglected in contemporary science, we will show that believing is a fundamental brain function on which individual and societal behavior is grounded.

## Introduction

This communication capitalizes on a new openness for understanding “religious phenomena” including “believing” as human abilities and activities ( Connors & Halligan, 2015; Krueger & Grafman, 2013). We address the neurophysiological and anthropological dimensions in the contemporary sciences of the largely neglected but nevertheless important phenomenon of believing. Explanations are proposed for the putative physiological and psychological implementations of the process in the human brain (i.e., where do beliefs come from?), and their functions (i.e., what are beliefs good for?). In sorting out the levels of building blocks and their functions, the neurophysiological processes underlying the behavioral process of believing as studied empirically in the individual are differentiated from more general belief system processes that operate in large collections of people such as communities and societies. This will lead to our hypothesis that there is an important relationship between what individuals believe and the processes by which they do so and the more over-arching belief systems in a society. Specifically, we will address hermeneutic, linguistic, behavioral and cognitive levels of explanation.

## A brief history of belief and believing

The issue of what a belief is and how beliefs are related to knowledge and rationality has been a fundamental issue in Western philosophy since the time of the great Greek philosophers Plato and Aristotle. It raises the fundamental question of how to best understand the relation between knowledge and belief ( Armstrong, 1973; Helm, 1999; Miller, 2013). Later, the importance of belief for understanding the role and meaning of Jesus Christ was highlighted by the writings of Saint Paul and other texts in the Christian Bible. Thus, in the history of Western thinking religious beliefs became more and more integrated into a dogmatic set of that what might be called “the Christian belief”.

After the Enlightenment, religious truth claims became suspect. By the 20 ^th^ century under the widespread influence of psychoanalytic theory ( Freud, 1928), religion was considered as an obsessional neurosis and “belief” negatively interpreted as a sign of human weaknesses. Thus, in psychology, religious phenomena including beliefs have from time to time been interpreted as deviant or at least as unneeded and subordinated under pathological labels such as neuroticism ( Hills *et al.*, 2004). A consequence is that there have been few attempts to empirically study the phenomenon of belief or to conceptualize “normal” belief on a scientific basis as opposed to its “pathological” manifestations in neuropsychiatric disorders ( Connors & Halligan, 2015; Pechey & Conners, 2012). Recently, there has been increased interest in the scientific discourse as well as in the general public in the nature of human belief. For example, in current psychology of religion there is increasing interest in belief and disbelief for both religious and atheist orientations ( Bullivant & Ruse, 2013; Schnell & Keenan, 2011) and in the relationship between religiousness and specific religious beliefs to spirituality and health ( Koenig *et al.*, 2012). This interest includes extensive and recently intensified debates about the meaning and utility of concepts such as faith, belief, transcendence, and spirituality ( Oviedo, 2017; Paloutzian & Park, 2013; Paloutzian & Park, 2015; Visela & Angel, 2017). In addition, in attempting to explore the neural correlates of religious experience, cognitive neuroscience implicitly implies that believing is a component of normal mental activity ( Azari *et al.*, 2001; Harris *et al.*, 2009).

Consequently, when we say holding a belief is a human ability, we mean that believing is envisioned as a mental activity generated by neural circuits in the brain ( Boyer, 2003). Thus, a belief is to be considered as a putative brain product of a believing individual and in general is entertained as a belief by humans. We hypothesize that beliefs serve a purpose in that they are linked to personal intuitive judgments about the subjective certainty of mental constructs and sensory perceptions, which is in line with the claims of others ( Harris *et al.*, 2008). Personal beliefs thereby function as part of the building blocks of intelligent behaviour ( Elliott *et al.*, 1995; Howlett & Paulus, 2015; Taves, 2015).

## The need for theory

To integrate findings such as the above into a coherent framework we need to formulate a theoretical concept of believing. This framework needs to comprise hermeneutic, linguistic, behavioral, and neurophysiological levels of explanation. In particular, in our attempt to develop an innovative understanding of believing and ultimately of _“_belief” we propose the following theoretical considerations.

A) In psychology of religion, belief is often understood as a religious phenomenon. However, a concept of “belief” does not equal religious belief because people believe all manner of things, most of which are a-religious. Thus “belief” has to be understood generically, not only religiously. Consequently, “belief” is a proper characterization and is relevant for secular and religious domains.
B) Because belief is a noun, it is often treated as a “state” ( Churchland & Churchland, 2013) or as an attitude towards someone or something, such as liking and favoring a person or disliking and seeing only the negative side of an issue. In contrast, we propose to understand “belief” as a mental activity generated by neural circuits in the human brain. Thereby we emphasize the process character of belief: it is not a state; believing is a mental process. When understood this way, the notion of belief can be dissociated from concepts with static meanings, which are usually expressed in substantive terms like “belief,” “faith,” or “spirituality”.
C) A model of the believing process has to account for inherent fluctuations of “beliefs” that people, nevertheless, perceive as rather static. How the fluidity of the believing process is related to our perception of belief stability has to be integrated into the model of believing. In addition to this itself being illuminating, it will provide a new approach to topics such as the “formation of belief” and maintaining “belief systems” ( Langdon & Coltheart, 2000).
D) A concept of a “physiological process of believing” has to integrate the complex notions of “time” and “process”. In philosophy, the understanding of time has been broadly reflected since antiquity and still is widely discussed ( Le Poidevin & McBeath, 1993), indicating that it cannot be reduced to merely “measuring” time or time being a “measurable” variable or property. Similarly, process thinking has a long tradition in Western philosophy, starting with Heraclitus` position “πάντα ῥεȋ“ (panta rhei; everything flows) and developed in the field of process philosophy as spawned in the writings of Bergson, Merleau-Ponty, and especially Whitehead indicating that process constitutes change and occurs through and interacts with time.
E) A full account of the believing process has to address the issue what determines putative starting and end points of such a process. We hypothesize that this explanation needs to include an account of the subliminal aspects of the process.

## From neuroscientific to anthropological dimensions of believing

Advances in the natural sciences have made it possible to scientifically explore to a certain degree virtually all physiological processes enabling human life. Analogous to the physical sciences, the life sciences seek to construct simplified models of biological processes that can be tested empirically by appropriate experiments. This approach was adopted by cognitive neuroscience with the aim of identifying fundamental processes underlying human behavior. We suggest that there are four levels of exploration in this enterprise.

A) There is a hermeneutic level rooted in philosophical issues. Cognitive neuroscience uses terms that come from different philosophical traditions and have their own history of meaning. We need to clarify which terms are most adequate to shape the theoretical paradigm of neuroscientific research and to reflect its findings. For example, the terms “process”, “relevance”, “imagination”, “meaning”, “value”, and “evidence” are less clear for cognitive neuroscience purposes (and, therefore, are shaped to indicate specific, technical meanings) as they might appear in everyday speech. Such attaching of technical meanings to everyday words is often necessary and a normal part of doing science.
B) The so-called linguistic turn in philosophy had sensible implications for the use of language. Even our everyday phrases such as “to think,” “to know,” or “to do” imply a connection to reality. We cannot avoid terms of this sort in this paper, but space constraints do not allow for philosophical discussion of their use. Suffice it to say that the terminology used in this field, especially when distinguished from the language of folk psychology, includes their relation to reality as a key aspect.
C) There is the behavioral level, which can be accounted for by heuristic cognitive models. By empirical research, models of this sort have been validated as, for example, the multimodal networks of memory, attention, and language ( Levelt, 1993; Mesulam, 1990).
D) Modern neurophysiological methods such as functional imaging and electroencephalographic techniques allow us to explore the temporal order in which different brain areas are engaged during performance of specific behavioral tasks. Studies of this sort have yielded models of brain function in terms of both plausibility and topographic and temporal realization in the human brain. An example is the review on the recently widely debated issue of “free will” ( Hallett, 2016).

In this communication we expand on the latter two topics and address the complexity of the psychological processes involved when individuals interact with their environment to understand what is going on around them. For our discussion we depart from the notion that from birth onwards humans have to learn to analyze signals coming from the environment in order to behave appropriately in response to them ( Seitz *et al.*, 2009). Also, they must develop insight into their own sensory capacities and bodily strength, and rely on them. These capacities are linked to the concept of a bodily and mental identity ( Sugiura, 2013) combined with self-esteem and a sense of agency ( Farrer & Frith, 2002; Northoff, 2011). This linkage allows people to retain a high degree of subjective certainty even when the situation is objectively unclear.

We can observe events at a behavioral level in the individual and in groups of individuals. But we have yet to learn what cognitive processes make for evaluative judgments at any level of specificity. Where do cognitive processes get the criteria they “use” to measure and evaluate internal and external stimuli? If we suppose the existence of a kind of “valuation system,” it may be best and intellectually sound to consider it as one of several aspects of a meaning system. This is because the concepts of measuring and valuing are intimately linked through meaning. For example, what has positive meaning is also valued, and what is valued is or has been measured, which affords its meaning for the person. In fact, all of the recent scholarship on meaning systems either explicitly or implicitly includes values or valuing among the list of components of a meaning system ( Markman *et al.*, 2013; Park, 2005). Therefore, just as global meaning systems are psychological structures comprised of interactive components that guide the interpretation of and response to information, so also are “valuation systems” a component of meaning systems that interact with all the other components of meaning systems and contribute importantly to the operation of the whole system.

People combine formal analytic and subjective affective judgments to arrive at propositions of the form “I believe that …”. We assume that the basic processes of believing are universal but are also modulated by human individuality. For example, it has been shown that individuals differ in how they detect and interpret noisy optical signals, and some might be prone to magical ideation. In fact, magical ideation might influence the judgement of contingencies ( Adelson, 1993; Brugger & Graves, 1997).

Recently, processes underlying believing have been labelled “credition” ( Angel, 2013a). Credition is a neologism based on the Latin verb *credere* (to believe). The notion of credition is different from faith, religion, and spirituality, and provides an empirical psychophysiological framework for the study of what believing is at the psychological, neuroscientific, and social levels of analysis. Doing this involves multilevel data mapping ( Paloutzian & Park, 2013; Paloutzian & Park, 2015) or bi-directionally “translating” the data and concepts from one level of analysis to an adjacent level of analysis in order to assess the degree to which they correspond. In outlining the heuristic model of credition, Angel & Seitz (2016) summarized it to include a number of cognitive and emotional operations affording believing. In particular, they presented correspondences between cognition, emotion, and credition operations and the neurophysiological processes of perception and valuation.

To understand the process of believing, it is essential to understand how people attribute personal meaning to specific sensory perceptions ( Paloutzian & Mukai, 2017; Seitz & Angel, 2014). Perception and attribution of value (valuation) are two dynamic and reciprocal processes that are at work simultaneously to enable this. Both of these neurophysiological processes have been studied extensively. Here, their major properties are highlighted as follows.

**Figure 1.**
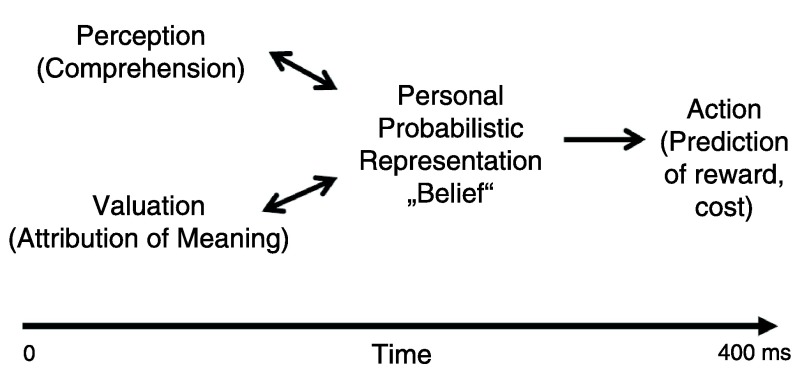
Mental operations underlying meaning making and guidance of behaviour in the probabilistic perception-action-valuation model (after Seitz *et al.*, 2009). Perception refers to the formal comprehension of external items and events; valuation affords the attribution of personal meaning to them. Both psychic functions operate in a dynamic and reciprocal fashion leading to personal probabilistic representations in the human brain. As the personal probabilistic representations are formed in milliseconds typically being implicit, they may become explicit owing to a high emotional loading and a repetitive exploration. The subsequent actions are based on the personal probabilistic representations being loaded with probabilistic predictions of reward and cost.

(1) Perception deals with the formal characteristics of physical stimuli experienced in the outside world (see Figure 1). The process employs sensory systems such as vision, audition, and somatosensation as well as higher order sensory information processing. The resulting representations comprehend feature identification, stereognosis, associations of pragmatic use, object-name associations, etc. or “Gestalt” as elaborated by von Weiszäcker (reviewed by Schott, 2014) and are stored in memory. This is a physical process that involves highly complex interactions between explorative movements and object perception and results in comprehending the object’s features ( Jeannerod, 1995; Roland & Mortensen, 1987). Internal mental states effectively represent external states in a probabilistic fashion ( Friston *et al.*, 2014). As illustrated in research on unstable picture puzzles, objects become more identifiable against a noisy background when either the signal-to-noise ratio or the duration of exposure increases ( Takei & Nishida, 2010). Moreover, the physical characteristics of objects are processed such that the perceived composition of the components of an object is matched against that of a previously perceived item ( Adelson, 1993). This process may distort the perception of the physical characteristics of the object but results in a meaningful “Gestalt“.

The speed with which sensory information is processed is important. Nervous tissue is exceptionally fast at doing so owing to the high excitability of nervous membranes, which allows for rapid inter-cell communication. High speed information processing is a phylogenetic advantage for control of behavior. Because of this high speed, most sensory information is processed non-consciously in the brain; only some bits of information enter awareness. Accurate material categorization of real-world images occurs as fast as 30 ms but increases in accuracy with longer exposure times, up to 120 ms ( Sharan *et al.*, 2014; van Gaal *et al.*, 2011). Thus, operationally, stimulation onset can be assumed to signify the start of the believing process. As soon as information reaches the primary visual cortex, early automatic processing in the 100ms range affects information transmission further downstream ( Nortmann *et al.*, 2015). This feed-forward processing has the feature of predictive coding. For example, visual information can guide predictive on-line scaling of hand aperture and strength of finger movements required for object grasping ( Diamond *et al.*, 2015; Pélisson *et al.*, 1986). Moreover, early responses in the amygdala (40 to 140 ms) are unaffected by attentional load, while later responses (280 to 410 ms) in the amygdala are modulated by attention ( Kuo *et al.*, 2009).

The perception of objects has many commonalities with the perception of events, but there are also clear differences ( Radvansky & Zacks, 2014). The most important difference is that objects are static entities, whereas events are fluid and evolve over time. Accordingly, events are perceived from a succession of patterns in which items of interest change over time in a coherent manner. Thus, an event becomes a meaningful percept by temporal coding. As an example, in the virtual reality environment the impression of a ball moving towards the observer is generated by the increasing size of the ball against a static background ( Cameirão *et al.*, 2010). Similarly, the movement of a ball between two persons in a virtual landscape generates the impression that the two people are throwing a ball to each other. Thus, the observer constructs a meaning and attributes it to the observed temporally evolving events. Other examples of temporal coding of events include the processes involved in writing, reading, and playing and listening to music. Again, decoding of the temporal sequence of single events provides the meaning of the events. In fact, the rapid temporal evolution of the electrical activity in the peripheral nerves recorded during hand writing can be played back to the nerves and shown to be capable of producing the same limb movements ( Aimonetti *et al.*, 2007).

(2) In addition, a dynamic and reciprocal process is concerned with processing the affective value of physical stimuli in the outside world and attributing person-specific meaning to them ( Figure 1). The personal probabilistic representations that result from these processes are typically implicit but can become explicit when the stimuli trigger high personal meaning ( Friston *et al.*, 2014). Prominent negative features of this sort are signals of potential harm or threat; prominent positive features may be signals of beauty or pleasantness ( Rolls, 2006). Such affective labeling is behaviorally highly relevant because it may evoke opposite motivations and responses, such as avoidance or desire. However, there are memory control mechanisms that become active 200 to 300 ms after stimulus onset allowing the individual to judge the stimulus as referring to the present reality ( Liverani *et al*., 2015). Thus, the subject’s memory is highly vulnerable towards subliminal distortions.

Objects of special relevance for humans are human faces, since facial expressions of emotion characterize interpersonal encounters and induce meta-analytic processes leading to the interpretation of the mental as well as the emotional state of the counterpart ( Potthoff & Seitz, 2015). As in the case of identification of other objects, these processes are fast; they take place within 40 ms, as is evident from behavioral and neurophysiological studies ( Bar *et al.*, 2006; Smith, 2012). In a more general sense, one may wonder how subjective categories such as aesthetic judgments become important. Pertinent to this issue, it has been shown that individual aesthetic preferences for faces are shaped by an environment associated with an individual -- not by genes, as found in judgments of attractiveness in over 570 monozygotic twins ( Germine *et al.*, 2015).

Finally, repeated experience with the same environmental objects or events stabilizes their cognitive-emotional representations so that, e.g., familiarity with an object or information promotes learning about it and increases a sense of trust in the object or information ( Chang *et al.*, 2010; d’Acremont *et al.*, 2013; Henkel & Mattson, 2011). In addition, representations already formed will be updated as new items and information are accommodated to the store of acquired knowledge. Also, there is recent evidence that learning is accompanied by subjective emotional loading. For example, learning invokes a sense of confidence that increases in proportion to the number of observations of a task as well as task performance ( Meyniel *et al.*, 2015).

Perceived information is what motivates the generating of actions ( Figure 1). Note that believing is inherent in this transformation such that knowledge acquired in the past and represented in probabilistic representations is linked to the future by probabilistic predictions. Generating actions involves intentions to act, action selection, inhibition of unwanted acts, and predicting reward and costs of acts ( Nachev *et al.*, 2008; Passingham *et al.*, 2010). In general terms, this refers to deciding what to do next. The neuroscientific basis for decision making has been shown to be related to reward valuation ( Grabenhorst & Rolls, 2011) and unconscious or intuitive selection that evolves within far less than 400 ms ( Chen *et al.*, 2010; Kahnt *et al.*, 2010; Schultze-Kraft *et al.*, 2016). The processes that regulate the performance of actions are replete with probabilistic reward and cost predictions determined by the personal meanings attributed to the mental representations of the signals from the outside world ( Friston *et al.*, 2014). The action-perception-valuation triad has been postulated to account, in context of a hierarchical dimension, for computations of physical, social, and cultural matters ( Sugiura *et al.*, 2015). The model proposed here binds these mental functions to a probabilistic neural code in a Bayesian sense. In the following we outline recent neurophysiological research showing the cerebral structures participating in these mental functions.

## Functional anatomy of the believing process

**Figure 2.**
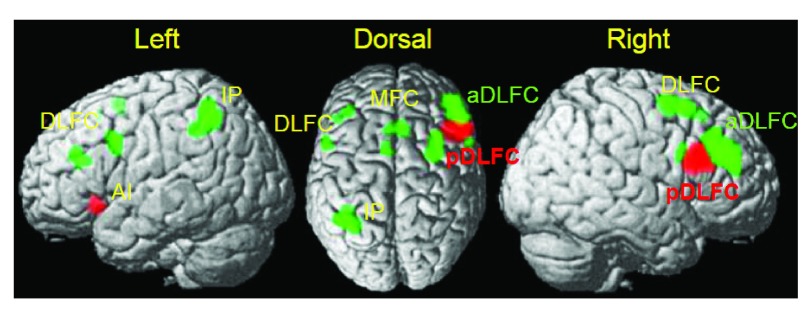
Non-overlapping cortical networks involved in decisions based on inferential beliefs about subliminal information. Red areas were functionally connected to activation of the posterior dorsolateral prefrontal cortex (pDLFC) during empathic evaluation in the pre-decision phase. The right pDLFC was connected with the left anterior insula (AI). Green areas belonged to a widespread network involving the anterior dorsolateral prefrontal cortex (aDLFC) which was activated related to verbal processing in the actual decision phase. The right aDLFC was connected to the right and left dorsolateral prefrontal cortex (DLFC), the mediodorsal prefrontal cortex (MFC), and the left inferior parietal lobule (IP). Note, that masking of the subliminal (40 ms) visual stimulus required the subjects to make a decision according to what they believed was the right answer. Further details in Prochnow *et al.* (2015).

Valuation of perceived information and making attributions intimately involves the medial frontal cortex ( Grabenhorst & Rolls, 2011; Kahnt *et al.*, 2010; Seitz *et al.*, 2006; & van Overwalle, 2009). This is also true for attributions about the mental states of others ( Bird & Viding, 2014; Kanske *et al.*, 2015). The dorsolateral prefrontal cortex is specifically involved in making attentive decisions ( Gray *et al.*, 2002; Niendam *et al.*, 2012). These psychological processes include valuation of delayed reward and engage extensive brain circuits including the medial and lateral prefrontal cortex ( Niendam *et al.*, 2012; Peters & Büchel, 2009; Thompson & Duncan, 2009). The activation of the dorsolateral prefrontal cortex during decision processes was correlated with gamma-activity in long-distance cortico-cortical synchronization and found to be related to the capacity of the working memory system and fluid intelligence scores ( Dehaene & Changeux, 2011; Federenko *et al.*, 2013; Roux *et al.*, 2012). There is recent experimental evidence that believing can also affect activity in these brain areas ( Howlett & Paulus, 2015; Ninaus *et al.*, 2013). As dopaminergic midbrain areas are tightly connected with these areas and encode shifts in beliefs, they may contribute to belief updating ( Schwartenbeck *et al.*, 2016). This means that the control of a person’s behavior is mediated by extensive neural networks that include the medial and dorsolateral prefrontal cortex related to comprehension of the formal sensory information and to emotional valuation of that information. As a model of the believing process we studied understanding other people’s behaviour in terms of most probable explanations using multidimensional functional magnetic resonance imaging (fMRI). The set-up of the experiment summarized in Figure 2 (an event-related fMRI study) allowed us to separate a pre-decision phase in which emotional information was presented, from the phase when the subjects were required to make their decision on verbal material. Since the information on which the decisions had to be based was presented only below the level of awareness, the subjects were put in a state of uncertainty. This is a situation in which it is typical for someone to rely on what he or she already believes to be correct.

The figure shows two different functional circuits including the brain areas described above. In addition, these circuits included the supplementary motor area (SMA) in the dorsal portion of the medial frontal cortex that provides the link to proactive movement control by adjusting the level of motor readiness affording response inhibition ( Chen *et al.*, 2010; Meder *et al.*, 2016) as well as free choice movement coding and behavioral tactics ( Matsuzaka *et al.*, 2016; Passingham *et al.*, 2010). Meta-analytic studies of functional neuroimaging data have revealed that different nodes in the medial frontal cortex are engaged in judgements of perceived formation and predictions of future events including the SMA and pre-SMA, ( Seitz *et al.*, 2006; van Overwalle, 2009). The neural hubs identified in these studies were arranged in a caudo-rostral gradient of increasingly more abstract information processing. As evident from resting state connectivity, the medial cortical areas and the parietal cortex were shown to be part of the so-called default mode perspective of humans ( Gusnard *et al.*, 2001).

Prospective valuation of rewards in a familiar context was reported to be related to activation of key nodes of emotional and autobiographical memory retrieval and dynamically modulated by frontal-striatal connectivity ( Sasse *et al.*, 2015). The modulation of cortical information by processing in trans-striatal relay loops has been described as of key importance for learning routines and rules as well as their combinations ( Graybiel & Grafton, 2015). Accordingly, the multiplex aspect of probabilistic cognitive-emotional representations involves cortical and subcortical networks. In effect, these data support the notion that believing in personal probabilistic representations is a normal brain function.

## Belief systems

The raw phenomenological mental representations in a person’s mind are not accessible to scientific exploration; thus their veridicality in the purest sense is neither provable nor disconfirmable. They constitute personal beliefs (i.e., meanings made) that can be characterized as falling into two general categories. First, there are beliefs that everyone in a group or society will hold, such as the belief that they all see an apple sitting on the table and that they can eat it and it will taste good. Beliefs of this sort have been addressed earlier and are subject to some degree of public empirical verifiability. Second, there are beliefs apparently unique to one person, such as someone being certain that he or she saw God and heard God’s voice – a report not subject to public verification. Either way, we engage in a functional inherent valuation process that involves focusing attention on the incoming information in a dynamic bottom-up-top-down fashion, the result of which forms our probabilistic accounts and beliefs about what is observed in the outside world ( Dehaene & Changeux, 2011; Wiese *et al.*, 2014). Thus, beliefs of individuals are created by mental processes that involve perception, attention, valuation, and storage as well as up-dating of information as described in detail in the previous part of this communication.

Given the above, it is obvious that belief-related language expressions are commonly used in everyday circumstances.. When someone says, “I believe God is the creator of the world,” he or she is stating a subjective proposition that cannot be empirically verified. Not uncommonly, the person becomes emotionally upset upon hearing others question or negate the statement. In contrast, the statement “I think this is so and so” expresses only a limited person’s certainty, while the statement “I believe this is so and so” expresses the person’s perspective that the observed fact or event has a higher degree of certainty on which he or she builds an emotional inclination to defend this stance. This is probably because people’s intuitive belief system appears to represent beliefs as either true or false rather than on an uncertainty gradient ( Johnson *et al.*, 2015). Moreover, beliefs activated by cues can profoundly affect behaviour, as has been found for gaze and other behaviors similar to those that respond to primes ( Kristjansson & Capana, 2010; Wiese *et al.*, 2014). Conversely, hypnotic suggestion can be seen as a form of inducing an imagination that is temporarily accepted as if it is believed, since hypnosis can exert profound changes on a person’s mood, thoughts, perceptions, and behaviour ( Halligan & Oakley, 2014). Consequently, we assume that personal probabilistic representations form the knowledge system of an individual displaying a high degree of momentary subjective relevance.

As people grow up and are imbedded in social groups, successful communication is fundamental to the exchange of meanings of perceptions, imaginations, and mental states. Thus, group evolution in addition to, not in place of, the evolution of individuals becomes important. Owing to the wealth of information to which each individual is confronted every day, information is communicated from person to person by language. Language is characterized by the human capacity to combine meaningful units into an unlimited variety of larger structures, each differing systematically in meaning. The capacity to generate a limitless range of meaningful expressions from a hierarchical structure of finite elements differentiates human language from all other animal communication systems ( Fitch & Hauser, 2004). This means that the most complete understanding of the processes of believing and communicating among humans requires that we examine the processes from micro to macro levels within a multilevel interdisciplinary paradigm ( Emmons & Paloutzian, 2003). In this instance, the intersection of anthropology and neuroscience can help us understand the relationships between the socio-cultural contexts in which people live and human brain function ( Keysers & Gazzola, 2010; Vogeley & Roepstorff, 2009) as will be outlined below.

**Figure 3.**
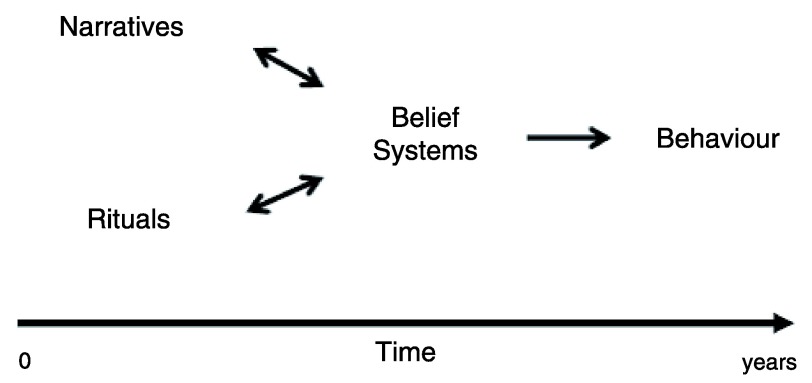
Formation of belief systems and their behavioral consequences as predicted by the probabilistic perception-action-valuation model. Narratives are socially transmitted in communities with many repetitions and ramifications in colloquial and formal settings enabling individuals to comprehend their contents. Similarly, socially practiced rituals allow for attribution of personal meaning to the belief systems. Importantly, subjects may be exposed to both sources repetitively over many years internalizing their meaning by dedicated and reciprocal psychophysical processes that lead to complex probabilistic representations, i.e. belief systems. These belief systems are similar among individuals who belong to the same social group or society. The subsequent behavior of the individuals is loaded with probabilistic predictions about their future, termed hope and fear.

Individuals are constantly faced with boundaries imposed by the surrounding people. There are universal dimensions of interpersonal and peer group social cognition that guide the individual’s behavior ( Fiske *et al.*, 2007). Thus, living in a society requires the generation of systems of probabilistic representations that are similar across individuals and exhibit a liability for communities as they give meaning to people’s collective work. As detailed by Schnell (2012), narratives typically taught implicitly and explicitly in families and schools provide the historical and identity-relevant background information for social groups, through passive listening as well as active reading and reciting. Thereby, narratives provide the formal content of belief systems ( Figure 3).

Narratives constitute a mental construct or meaning for the history of a community or society as well as for occasions of festive events throughout the year. People may be repeatedly exposed to a narrative (e.g., at annual special events); this affords them an opportunity to comprehend their meaning and learn them by heart. These narratives are typically a religion but can be secular as observed in modern societies. Acceptance of the narratives is strengthened as people participate in group rituals, which involve defined actions whose performance within a community or society leads to highly predictive experience by their members ( La Cour & Hvidt, 2010; Seligman & Brown, 2010). Through such group activities, emotional value and personal meaning is attributed to the belief systems that are shared by the corresponding groups of people and societies ( Figure 3). Looked at from a more molecular level of analysis, the psychophysical and neurophysiological processes affording the internalization of narratives and rituals in the individuals have been summarized above. Accordingly, _“_religious beliefs“ and _“_secular“ beliefs“ are hypothesized to be brought about by similar, if not identical processes of believing but differ by their specific contents conveyed by narratives and rituals.

People in communities and societies may be similar in their belief systems due to exposure to virtually identical narratives and rituals. since rituals are likely to bias humans to accept narratives as beliefs which are held in social groups and secular and religious faith groups. Thus, rituals play a key role in stabilizing beliefs owing to their standardized practice and regular repetitions at a present moment or more importantly at regular times each year. This is mediated by high fidelity imitation that mediates conventional learning ( Legare & Nielsen, 2015). Because rituals are rooted in narratives or myths that refer to the past, even beyond the limits of personal experience, their regular repetition produces the feeling of familiarity, high predictability, reward and transcendence. They can constitute the experience and knowledge and, thereby, the belief systems of individuals from childhood onwards ( Seligman & Brown, 2010). With this background, the individual’s experience gets linked to narrative knowledge through instruction and/or associative learning – which is extremely powerful because it may take only one to two repetitions ( Blechert *et al.*, 2016). In addition, the combination of the verbal and pragmatic information generates trust in the promise provided by the narratives, strengthening people against perceived threats, even to their physical integrity and prosocial behavior ( Boyer & Liénard, 2006; Talmont-Kaminski, 2016). Ultimately, it leads to the inference of moral and ethical standards that are derived from such narratives and limits the possible actions to be selected ( Mesulam, 2008).

Nevertheless, belief systems have a personal aspect that reflects the experience, attitudes, and personality of the individual. As experience changes over time, belief systems are likely to change as expectations based on past experience are violated by novel experience ( Angel & Seitz, 2017). In addition, each individual has a unique intuitive pre-evidential and probabilistic judgement of the world. For example, intuitive beliefs may originate from the naive but nevertheless fundamental dualistic experience of the surrounding immanent physical world and the seemingly immaterial sky. They can be a powerful component of an individual’s belief system. Belief systems include the individuals’ implicit or explicit answers (or attempts at such) on how to cope with the future, and how to provide existential meaning whether secular, spiritual, or religious ( La Cour & Hvidt, 2010). They also address other issues such as what values to hold, what priorities to live by, and what is ultimately most important. The predictions for the future are probabilistic; they may provide hope of reward or fear of punishment depending on how one has lived ( Figure 3). Also, depending on the promises of a particular belief system, the individual may be in a position to anticipate his or her future in a way most suited to his or her preference and to base their behavioral decisions hereon. At a neurophysiological level, it was shown that the cultural self-construal mind-set involves parieto-frontal brain areas including the medial frontal cortex as described above for spontaneous evaluation and behavioral control ( Leuthold *et al.*, 2015; Wang *et al.*, 2013).

In this scientific examination of believing and belief systems, several distinctions in the meanings of key terms must be kept in mind, for example, in addition to “belief” too often assumed to connote something religious or spiritual, it has also too often been assumed that issues of belief do not concern people who are nonreligious or generically secular ( Stich, 1996). Sometimes it is assumed that they don’t have any beliefs ( Bullivant & Ruse, 2013). But social science research documents that believing abounds in such persons even though the content differs from that in typical religious beliefs ( Schnell & Keenan, 2011). Further, the meanings of specific terms should be meticulously teased apart in order to avoid confusion. For example, because “religion” is not one thing but many, it is better to talk about specific religions, because almost no statement about what “religion” does will hold for all of them as elaborated elsewhere ( Paloutzian & Park, 2013). And it is circular to define religion with reference to “the sacred” or as the “search for significance in ways related to the sacred” ( Pargament, 1992) because anything can be significant (i.e., matter to someone) and literally anything, including a rock, idea, or war, can be attributed the property of sacrality. Above that it is important to acknowledge that “religiosity” and “religion” are not the same as detailed elsewhere ( Angel & Seitz, 2016). For example, various and even contradictory expressions of religiosity may be associated with a given religion. Moreover, “religiousness” connotes the processes that mediate how one appropriates and manifests one’s religion in life, not “the religion” as such (whatever “the religion” might “really” mean).

Theoretically, “religious experience” cuts across most of the above constructs, in addition to being manifest in both the individual and collective realms. But “experience” is a phenomenologically private sphere. It is not a matter of public knowledge even though the claims, words, and behaviors associated with purported experiences are. For reasons such as these, “ *religion*” explains little about how individuals make use of religion when fostering their “religiosity” ( Angel, 2013b). For instance, some may integrate their religion into their worldview in a more peaceful and harmonious way, while others may do it in a more aggressive or aversive manner. Likewise, for some, adopting one specific version of one religion is the key to this life and a life in the hereafter, whereas for others anything or nothing will do just fine. For some, William James’ (1985) emphasis on “religious experience” constitutes the defining moment of a life. In contrast, it has recently been argued that myths, rituals or transcendent experience can constitute “implicit religiosity” ( Schnell, 2003) and that there are “born believers” ( Barrett, 2012). These ideas suggest that humans come automatically equipped to engage in the process of believing many things -- whether secular or religious, or ordinary and mundane vs. lofty and idealized. That such objects of belief become incorporated into over-arching belief systems is consistent with the accumulating empirical evidence that the human proclivity toward worldview construction can be conceptualized as a by-product of normal human cognitive processes ( Boyer, 2003; Kapogiannis *et al.*, 2009).

Overall, we are beginning to understand one of the most fundamental processes that enable humans to be human – the process of believing. We suggest that the mental processes described in this paper represent fundamental human brain functions that transform cognitive and emotional perspective taking into accounts of personal perspective making, i.e., into views of secular and non-secular transcendence. One limitation of the concept of believing as presented here is that it is rooted in Western thinking, especially in the English language. Although beliefs and believing can have different connotations in various religions and cultural environments ( Angel & Seitz, 2016), our model can nevertheless generate a diversified but collaborative discussion on how to relate empirical data to science-based models of believing.
