# Supplementary material for: Processes of believing: Where do they come from? What are they good for?
Source: F1000Res. 2017 Jan 17;5:2573. Originally published 2016 Oct 25. [Version 2] doi: 10.12688/f1000research.9773.2 (PMC5200943; doi:10.12688/f1000research.9773.2)
Supplement: Peer review history

# Peer review: Referee response for version 1

**DOI:** 10.5256/f1000research.10535.r18600

**Published:** 2016-12-19

**Authors:** Tatjana Schnell, Roberto Viviani

**Affiliations:** 1 Institute of Psychology, University of Innsbruck, Innsbruck, Austria; 2 Innsbruck University, Innsbruck, Austria; 3 Psychotherapy Clinic, University of Ulm, Ulm, Germany

The authors provide a broad introduction to a novel theoretical concept, the process of believing. It is complemented by references to empirical findings that lend support to the theoretical claims. The concept’s usefulness for neuroscience and anthropology, in particular, is discussed.

 As the authors note, the act, or process, of believing has largely been ignored by scholars. While psychology offers insights into both cognition and emotion, believing seems not to be well covered by these theories. The authors suggest defining processes of believing as probabilistic representations, associated with specific personal meaning and value. Rooted in neural processes, processes of believing are proposed to guide human behaviour.

 The endeavour is topical and worthwhile, since much of our proposed knowledge is actually belief, and clear differentiation between both will be fruitful. Furthermore, belief is all too often associated with the realm of the supernatural, and thus viewed as something that is only of concern for studies of religion, etc. But believing is widespread, also in mundane everyday life, as the authors suggest.

 In some places, the text might benefit from more coherence. In the first section, e.g., we had the sense that the provided list of definitions is of no consequence for the rest of the manuscript.

 Due to the novelty of the research subject, the terminology is of special importance. The authors call “normal belief” a “brain product” linked to personal intuitive judgements about the subjective certainty of mental constructs and sensory perceptions. The term “normal belief” might not be the best choice of term, since it seems to refer to something normative, or some belief held by the majority of people (thus being normal).

 The reader is also left wondering about the relationship between “belief” as in “predictions about the next event”, or judgments where religious issues play no role, and religious belief. If the purpose of the term “normal belief” is to root religious belief into belief as a more general cognitive process, it should be renamed accordingly and more explicitly.

 The process of perception is described as resulting in “comprehending the object’s features” (p. 4), and, later, as resulting “in a meaningful illusion” (p. 4). The two descriptions seem to contradict each other; instead of calling the outcome an illusion, it might better be viewed as a “Gestalt”, i.e. a meaningful perception that provides clues for action to follow ^1^^,^^2^.

 Starting from page 4, the authors review neural circuits of stimulus perception, stimulus valuation (including its emotional tone) and decision making. The authors propose that this circuit underlies belief formation ("personal probabilistic representations").

 It should be noted that the neuroscience they refer to here deals with predictions about events whose valuation, and consequent motivational effects, are entirely utilitarian ^3^^,^^4^. The purported computational processes associated with these networks concern prediction of rewarding or aversive outcomes (probabilistic predictions of reward or fear of punishment, p. 8). This leaves the reader wondering if this has the consequence that there is nothing specific about religious beliefs, relative to other utilitarian or adaptive cognitive-behavioural strategies. The ventromedial prefrontal cortex, where, as the authors note, complex properties of stimuli ^5^^-^^8^ or the context of their presentation ^9^ may be integrated to compute stimulus valuation and its emotional quality ^10^ has been touted as the neurological machinery that allows consistent decision making, in effect computing utilities as in the economic understanding of this term ^4^^,^^11^.

 It is interesting to conjecture that these same networks could be the starting point of beliefs, but the evidence does not imply this in any straightforward manner. If predictions are beliefs also Pavlov’s dog was ‘believing’ after hearing the bell. Importantly, one may not be conscious of anticipating anything but still bear the motivational consequences of these unconscious predictions ^12^, as when presented with conditioned cues subliminally. An issue that seems to be relevant in this respect is that these networks are very likely not monolithic (as the authors themselves note in commenting Figure 2), but are thought to involve parallel valuation and decision mechanisms, associated with different strategies to process the inputs and compute response ^13^. But then one would expect the notion of belief to be explicated in terms that are more specifically relevant for some, but not all, of these mechanisms.

 Previous functional neuroimaging of generic beliefs ^14^^-^^15^ or of beliefs that are more specifically religious ^16^ raise the issue of whether cognitive decision making bears the traces of different strategies to arrive to a response, associated with different brain networks. This may be particularly relevant for beliefs, as there is good reason to single out those cognitive decisions that are marked by bias. The ventromedial prefrontal cortex seems again to play an important role in this respect ^11^^,^^14^^,^^17^, as an important hypothesis is that beliefs may be a specific class of intuitive, pre-evidential judgments, demonstrably produced when a specific part of the valuation-decision making network gains the upper hand. The relationship of religious belief with biased judgment seems important, but is an issue that the manuscript leaves entirely open.

 Several findings also implicate the amygdala in choices where emotions and intuitions play an important role ^17^^,^^18^. Considering the role of neural substrates directly linked to emotion when choosing between sources of meaning could add to the bigger picture of how acts of believing are linked to non-rational processes. Decision making between potential sources of meaning such as religion/spirituality, nature, community, may be related to the affective network in the brain, but not to more refined goal-directed mechanisms in the prefrontal cortex, as is suggested by a recent study ^19^.

 On a psychological level, further construct clarification could ensue from discussing relationships between processes of believing and intuition. Research findings on intuition ^20^ might help to better understand processes of believing, and to identify the boundaries between both.

 Minor points:

 Schnell, 2003 ^21^ is quoted as suggesting that “humans are born with an implicit religiosity” (p. 6). This seems to be a misreading of the publication, which aims to clarify in which cases implicit religiosity is present: “All those contents which are structured as myth, ritual or transcendent experience, and evaluated as meaningful by an individual, can be said to constitute implicit religiosity“ (p. 89). Not every person is implicitly religious, and it is not an inborn characteristic – although the propensity to elevate what is personally meaningful, and to express it by means of stories, rituals, and extraordinary experiences that go beyond the rational, appears to be common to all humans.

 Why does a process of believing necessarily have to have a start and a closure, as suggested by the authors?

We have read this submission. We believe that we have an appropriate level of expertise to confirm that it is of an acceptable scientific standard.

## References

[1] SchnellT: The Sources of Meaning and Meaning in Life Questionnaire (SoMe): Relations to demographics and well-being. *The Journal of Positive Psychology*.2009;4(6) : 10.1080/17439760903271074 483-499 10.1080/17439760903271074

[2] SchnellT: Psychologie des Lebenssinns. *Springer*.2016;

[3] RangelACamererCMontaguePR: A framework for studying the neurobiology of value-based decision making. *Nat Rev Neurosci*.2008;9(7) : 10.1038/nrn2357 545-56 10.1038/nrn2357 18545266PMC4332708

[4] LevyDJGlimcherPW: The root of all value: a neural common currency for choice. *Curr Opin Neurobiol*.2012;22(6) : 10.1016/j.conb.2012.06.001 1027-38 10.1016/j.conb.2012.06.001 22766486PMC4093837

[5] O'DohertyJP: Lights, camembert, action! The role of human orbitofrontal cortex in encoding stimuli, rewards, and choices. *Ann N Y Acad Sci*.2007;1121: 10.1196/annals.1401.036 254-72 10.1196/annals.1401.036 17872386

[6] ChibVSRangelAShimojoSO'DohertyJP: Evidence for a common representation of decision values for dissimilar goods in human ventromedial prefrontal cortex. *J Neurosci*.2009;29(39) : 10.1523/JNEUROSCI.2575-09.2009 12315-20 10.1523/JNEUROSCI.2575-09.2009 19793990PMC6666137

[7] SmithDVHaydenBYTruongTKSongAWPlattMLHuettelSA: Distinct value signals in anterior and posterior ventromedial prefrontal cortex. *J Neurosci*.2010;30(7) : 10.1523/JNEUROSCI.3319-09.2010 2490-5 10.1523/JNEUROSCI.3319-09.2010 20164333PMC2856318

[8] LimSLO'DohertyJPRangelA: Stimulus value signals in ventromedial PFC reflect the integration of attribute value signals computed in fusiform gyrus and posterior superior temporal gyrus. *J Neurosci*.2013;33(20) : 10.1523/JNEUROSCI.4809-12.2013 8729-41 10.1523/JNEUROSCI.4809-12.2013 23678116PMC3865515

[9] WilsonRCTakahashiYKSchoenbaumGNivY: Orbitofrontal cortex as a cognitive map of task space. *Neuron*.2014;81(2) : 10.1016/j.neuron.2013.11.005 267-79 10.1016/j.neuron.2013.11.005 24462094PMC4001869

[10] RoyMShohamyDWagerTD: Ventromedial prefrontal-subcortical systems and the generation of affective meaning. *Trends Cogn Sci*.2012;16(3) : 10.1016/j.tics.2012.01.005 147-56 10.1016/j.tics.2012.01.005 22310704PMC3318966

[11] PlassmannHO'DohertyJRangelA: Orbitofrontal cortex encodes willingness to pay in everyday economic transactions. *J Neurosci*.2007;27(37) : 10.1523/JNEUROSCI.2131-07.2007 9984-8 10.1523/JNEUROSCI.2131-07.2007 17855612PMC6672655

[12] LeDouxJE: Coming to terms with fear. *Proc Natl Acad Sci U S A*.2014;111(8) : 10.1073/pnas.1400335111 2871-8 10.1073/pnas.1400335111 24501122PMC3939902

[13] DawNO’DohertyJ: Multiple Systems for Value Learning.2014; 10.1016/B978-0-12-416008-8.00021-8 393-410 10.1016/B978-0-12-416008-8.00021-8

[14] GoelVDolanRJ: Explaining modulation of reasoning by belief. *Cognition*.2003;87(1) :B11-22 1249910810.1016/s0010-0277(02)00185-3

[15] HarrisSShethSACohenMS: Functional neuroimaging of belief, disbelief, and uncertainty. *Ann Neurol*.2008;63(2) : 10.1002/ana.21301 141-7 10.1002/ana.21301 18072236

[16] HarrisSKaplanJTCurielABookheimerSYIacoboniMCohenMS: The neural correlates of religious and nonreligious belief. *PLoS One*.2009;4(10) : 10.1371/journal.pone.0007272 e0007272 10.1371/journal.pone.0007272 19794914PMC2748718

[17] De MartinoBKumaranDSeymourBDolanRJ: Frames, biases, and rational decision-making in the human brain. *Science*.2006;313(5787) : 10.1126/science.1128356 684-7 10.1126/science.1128356 16888142PMC2631940

[18] SeymourBDolanR: Emotion, decision making, and the amygdala. *Neuron*.2008;58(5) : 10.1016/j.neuron.2008.05.020 662-71 10.1016/j.neuron.2008.05.020 18549779

[19] VivianiRDommesLBeschonerPStinglJSchnellT: Choosing between human values and food: the Pavlocian substrates of intrinsic preferences. *14th Annual Conference of the Society of Neuroeconomics*.2016;

[20] GigerenzerG: Gut feelings: The intelligence of the unconscious. *Penguin*.2007;

[21] SchnellT: A Framework for the Study of Implicit Religion: The Psychological Theory of Implicit Religiosity. *Implicit Religion*.2007;6(2) : 10.1558/imre.v6i2.86 10.1558/imre.v6i2.86

# Response

**Published:** 2017-01-06

**Authors:** Rudiger Seitz

**Affiliations:** Centre of Neurology and Neuropsychiatry, Department of Neurology, Heinrich-Heine-University Düsseldorf, Germany

We thank the reviewers for their constructive comments and thoughtful questions concerning a number of aspects in our manuscript that stimulated us to engage in further clarifying these points. We will detail successively the corresponding changes we made in the manuscript as follows:

 As suggested we reviewed the first section carefully and rephrased it to enhance the visibility of coherence within the section and with respect to the rest of the paper.

 We explain that the notion of "normal belief" is usually contrasted to its pathological manifestations in brain diseases (page 4). In accordance with the reviewer dropped this expression from the manuscript, since it may cause associations mentioned by the reviewers which were not intended.

 We now are more specific concerning the questioned relation of "belief" and religious belief. We do not want to expand on the adjective "religious" which is ambiguous as we have outlined elsewhere (Angel and Seitz 2016). With respect to the question of the reviewer and for improved clarity we transposed paragraphs in the final section and now state on page 20 of the revised manuscript that "religious belief" in the sense of "non-secular belief" and "secular belief" are hypothesized to be brought forward by similar, if not identical processes of believing but that they differ by their specific contents. In fact, they differ by narratives as we state on page 19. Please, note that believing is considered a fundamental brain process entirely separate from religious beliefs as stated explicitly on page 20.

 We are entirely happy with the suggestion of the reviewers to refer to "Gestalt". This notion was developed by von Weizsäcker in the 40ies of last century. To his work we now refer in the revised manuscript on page 11.

 With respect to the discussion about prediction we would like to point out that believing and beliefs should not be reduced to prediction. We now state on page 14 that believing pertains to experience, i.e. knowledge acquired in the past. Moreover, we outline that believing links the past to the future as it allows the individual to make predictions (pages 12, 14). For example, acquired knowledge and predictions work on the physical world being entirely probabilistic. This becomes apparent by the automatic unconscious servo control of the hand for object grasping (Diamond et al, Pélisson et al; not cited on page 12). Likewise the meaning a subject attributes to an object or event reflects his/her prior experience with such an object or event providing a basis for estimating its future implication; specifically, the question is whether the object or event is beneficial or satisfying for the subject or adverse or deleterious (page 14). Finally, decision making is consistent only as long as a belief has not been modified in response to new and violating information. This topic is explained in greater detail elsewhere (Angel and Seitz 2017) now cited on page 21.

 The comment about Pavlows reflex is noteworthy. As the reviewer points out, this is a subconscious process that cannot be influenced voluntarily by the subject. And certainly the onset of salivation induced by the conditioning ringing of a bell reflects an automatic prediction. We can exaggerate this argument extending it to all types of reflexes including the eyelid reflex and the muscle tendon reflexes: the neuronal machinery operates with the goal to protect the integrity of the organism against predicted harm or to prepare it optimally for an upcoming action. If we accept this, we may want to argue that even the basic neural circuits are equipped with a machinery that internalizes sensory experience to code for appropriate behaviour for integrity and survival of the organism. Higher cognitive and emotional functions that constitute believing are more complex working in parallel cortico-cortical and cortico-subcortical circuits in the human brain. These complex brain systems are suited to afford the integrity and survival of the individual human and of groups of humans including societies as we now state on page 21.

 We agree that beliefs are based on intuitive pre-evidential and probabilistic judgments. We now state on page 20 that rituals are likely to bias humans to accept narratives as beliefs held in social groups as well as in secular and religious faith groups.

 Decision making is a brain function following and engaging judgments and predictions. In other words, decision making is down the road with respect to believing. We absolutely agree that different types of information processed in parallel and highly interconnected brain circuits come into play. The point we would like to make, however, is that the interface for such complex self-oriented computations seems to be the medial prefrontal cortex that comprises multiple functional units as evident from Figure 2 and outlined in greater detail elsewhere (Seitz et al. 2006, van Overwalle 2009). We now emphasize this issue on page 16 and refer to it later in the manuscript again (page 21).

 We are grateful about the clarification concerning the notion of implicit religiosity and modified this phrase accordingly (page 23).

 In our empirical model we use the novel sensory stimulus as starting point of the believing process and the establishment of a probabilistic representation as the endpoint of the believing process. This has now been stated clearly on page 11. However, as write on page 21 new experiences constantly entertain an update of such a representation reflecting the potentially fluid nature of beliefs.

# Peer review: Referee response for version 1

**DOI:** 10.5256/f1000research.10535.r17817

**Published:** 2016-11-21

**Authors:** Peter W. Halligan, Michael H. Connors

**Affiliations:** 1 Learned Society of Wales (LSW) , Cardiff, UK; 2 Department of Cognitive Science, Macquarie University, Sydney, NSW, Australia

In this paper, the authors discuss the notion of belief and its explanatory role across a wide range of academic disciplines. These include, for example, psychology, religion, philosophy, anthropology, and cognitive neuroscience. The authors aim to demonstrate that believing is a core function of the brain that guides much of our behaviour. They also argue that these cognitive processes operate in narratives and rituals across both religious and secular contexts. To address these goals, the authors provide a brief, if sometimes dense, discussion of belief, which includes the history and philosophy of the concept, the role of belief in anthropology and neuroscience, and belief’s more specific functions in religion, rituals, and narratives.

 The paper provides an interesting contribution to a historically neglected area of psychology and cognitive neuroscience, namely understanding and characterising the nature of belief. That said, the paper is perhaps too ambitious in scope, covering many different topics – including a conceptual history of belief and the role of belief in areas such as philosophy, religion, anthropology, perception, and cognitive neuroscience. Consequently, the paper at times appears a little unfocused and difficult to follow. It could be improved by having a clearer structure and possibly outlining how the various sections contribute to the central goals of the paper. Given the breadth of coverage, many of the topics cannot be covered in the detail they deserve. One could argue, given the authors’ central argument, that much of the historical review – including the sections on the history and philosophy of belief – could be omitted without detracting from the main purpose of the paper.

 The main focus of the paper remains the anthropology and cognitive neuroscience of belief. The authors here outline four levels of explanation for understanding belief – hermeneutic, linguistic, behavioural, and neurophysiological. These levels of explanation are not, however, always fully justified. It is not clear, for example, what the distinction between the hermeneutic and linguistic is and also whether the behavioural level can be necessarily grouped with the cognitive. It also remains unclear how the levels of explanation inform the rest of the paper.

 In one particular section, the authors discuss the importance of perception. Specifically, they note “ *To understand the process of believing, it is essential to understand how people attribute personal meaning to specific sensory perceptions*” (p. 4). The authors then provide a description of the processes involved in perceiving the physical characteristics of stimuli and the processes involved in attributing affective value and meaning. This important section could be expanded to include more evidence in support of the claims being put forward. In its current form, it is unclear how much is intended as a description of fact and how much involves the authors’ own theoretical take.

 The section on neuroscience is also important to the authors’ central thesis. The authors outline a model of believing as involving different brain regions. As this is possibly one of the most novel and significant parts of the paper, this section would benefit by being expanded to provide more details. For example, the authors make several claims about the role of different brain regions for selective cognitive functions (e.g., they suggest that valuation of perceived objections and attributions involves the medial frontal cortex) and then cite papers without much further explanation. The authors should engage more with the literature that they cite and explain how the research support their claims. They could also outline their model more clearly and how it relates to belief overall (rather than just sub-component processes, like the valuation of percepts).

 A further issue in these sections that could be addressed is that it is not clear whether the authors reach their overall goal. They state in the paper’s abstract: “ *We present evidence suggesting that believing is a human brain function which results in probabilistic representations with attributes of personal meaning and value and thereby guides individuals’ behaviour*” (p. 1). However, much of these sections on perception and neuroscience is descriptive and, as already noted, relies on citations of previous papers without further explanation of how these support their claims. The authors could more specifically highlight what they consider to be evidence for their model and discuss more critically how it supports their account.

 In the final section, the authors turn to belief systems in religion, rituals, and narratives. The authors here suggest that similar belief processes are common across both religious and secular contexts. It is not clear, however, why people would doubt this idea in the first place. The discussion, like other sections, contains some interesting material, but again is limited somewhat by selective engagement with existing literature. For example, the authors suggest “ *because ‘religion’ is not one thing but many, it is better to talk about specific religions, because almost no statement about what ‘religion’ does will hold for all of them*” (p. 6), then appear to make general claims about religion, religiosity, and religious experience. Apart from this apparent inconsistency, it is unclear whether this is a consensus opinion by all religious scholars. Other minor examples include claims about how “ *humans are born with an ‘implicit religiosity’*” (p. 6) and that rituals “ *constitute the experience and knowledge and, thereby, the belief systems of individuals from childhood onwards*” (p. 7), where it is similarly unclear how established these claims are.

 In sum, the paper addresses an important and timely topic, and presents proposals for a potentially novel perceptual/neural model for understanding belief. The paper, however, would benefit by focusing and clarifying its underlying purpose and reducing the breadth of coverage. The paper could also be improved by discussing the evidence and arguments supporting their central claims in greater detail.

We have read this submission. We believe that we have an appropriate level of expertise to confirm that it is of an acceptable scientific standard, however we have significant reservations, as outlined above.

# Response

**Published:** 2017-01-06

**Authors:** Rudiger Seitz

**Affiliations:** Centre of Neurology and Neuropsychiatry, Department of Neurology, Heinrich-Heine-University Düsseldorf, Germany

We appreciate the constructive comments of the reviewers which helped us to revise our manuscript as we explain in the following point-to-point response.

 According to their suggestion we rephrased the Introduction to outline more clearly how the various sections contribute to the goal of this paper. Furthermore, the section about the history and philosophy of belief was shortened and rephrased with a couple of additional citations to highlight our standpoint.

 We now have introduced further explanations to make clear the different levels of exploration such as the hermeneutic, linguistic, behavioral and the cognitive level to define the process of believing (page 5).

 Following the suggestions of the reviewers we have expanded our description about the physiological basis of perception and probabilistic coding (pages 12, 13, 14). Also, we now spell out more clearly our standpoint to this theoretical matter (page 10). Admittedly, however, we can only sketch out the topics relevant for our discussion (page 14), while a comprehensive review of the literature would go beyond the limits of this opinion paper.

 We substantiated the relation of the references to our manuscript and describe that a number of physiologically well described processes are essential for the processes of believing. We do not claim that they are exclusive. But in our view empirical evidence provides the framework for their contribution to the processes of believing as we now state explicitly on pages 5.

 We have rephrased the criticized statements about religion, religiosity and religious experience (page 20). Also, we introduced appropriate references in the final section to substantiate our claims.

# Peer review: Referee response for version 1

**DOI:** 10.5256/f1000research.10535.r17438

**Published:** 2016-11-08

**Authors:** Motoaki Suguira

**Affiliations:** 1 Institute of Development, Aging and Cancer (IDAC), International Research Institute of Disaster Science (IRIDeS), Tohoku University , Sendai, Japan

This is a good comprehensive review and model of the scientific status of the religious and secular belief or believing process.

 Major point:

- It would be more helpful for the readers if there was emphasis on what is the key aspects of the current model that makes it new and different from previous models of the authors or other researchers.

Minor point:

- While the two figures have very similar format, in the figure 1 there is a specific quantity of the time (i.e. 40ms) but not in the figure 2; the former looks a kind of experimental data and the latter a conceptual schema. It may be better to clarify whether two figures are similar or very different in terms of the roles in the manuscript (i.e. data or schema).

I have read this submission. I believe that I have an appropriate level of expertise to confirm that it is of an acceptable scientific standard.

# Response

**Published:** 2017-01-06

**Authors:** Rudiger Seitz

**Affiliations:** Centre of Neurology and Neuropsychiatry, Department of Neurology, Heinrich-Heine-University Düsseldorf, Germany

We thank the reviewer for his comments and now make clear on page 14 what the specific aspects of our model of believing are as compared to previous accounts.

 We assume that for some reason the time scale was not in the print-out of Figure 3. We absolutely agree that a time scale is important for this figure, since in contrast to Figure 1 the scale goes over years. We respectfully submit that the scale is shown in the figure.
